# Metabolomic Changes as Key Factors of Green Plant Regeneration Efficiency of Triticale In Vitro Anther Culture

**DOI:** 10.3390/cells12010163

**Published:** 2022-12-30

**Authors:** Renata Orłowska, Jacek Zebrowski, Wioletta Monika Dynkowska, Piotr Androsiuk, Piotr Tomasz Bednarek

**Affiliations:** 1 Plant Breeding and Acclimatization Institute-National Research Institute, Radzików, 05-870 Błonie, Poland; 2 Institute of Biology and Biotechnology, University of Rzeszow, Al. Rejtana 16c, 35-959 Rzeszow, Poland; 3 Department of Plant Physiology, Genetics and Biotechnology, Faculty of Biology and Biotechnology, University of Warmia and Mazury in Olsztyn, 10-719 Olsztyn, Poland

**Keywords:** androgenesis, β-glucans/pectins, copper, glutathione, S-adenosyl-L-methionine, regeneration efficiency, triticale

## Abstract

Green plant regeneration efficiency (GPRE) via in vitro anther culture results from biochemical pathways and cycle dysfunctions that may affect DNA and histone methylation, with gene expression influencing whole cell functioning. The reprogramming from gametophytic to sporophytic fate is part of the phenomenon. While DNA methylation and sequence changes related to the GPRE have been described, little attention was paid to the biochemical aspects of the phenomenon. Furthermore, only a few theoretical models that describe the complex relationships between biochemical aspects of GPRE and the role of Cu(II) ions in the induction medium and as cofactors of enzymatic reactions have been developed. Still, none of these models are devoted directly to the biochemical level. Fourier transform infrared (FTIR) spectroscopy was used in the current study to analyze triticale regenerants derived under various in vitro tissue culture conditions, including different Cu(II) and Ag(I) ion concentrations in the induction medium and anther culture times. The FTIR spectra of S-adenosyl-L-methionine (SAM), glutathione, and pectins in parallel with the Cu(II) ions, as well as the evaluated GPRE values, were put into the structural equation model (SEM). The data demonstrate the relationships between SAM, glutathione, pectins, and Cu(II) in the induction medium and how they affect GPRE. The SEM reflects the cell functioning under in vitro conditions and varying Cu(II) concentrations. In the presented model, the players are the Krebs and Yang cycles, the transsulfuration pathway controlled by Cu(II) ions acting as cofactors of enzymatic reactions, and the pectins of the primary cell wall.

## 1. Introduction

Numerous cytological and genetic studies have shown that triticale, an artificial crop created around 120 years ago that combines the rye and wheat genomes, continues to be genetically unstable. Its instability was also noted for in vitro tissue culture [1], where chromosome modifications, including aberrations, deletions, and insertions were detected as well as DNA methylation patterns were found [2]. The species is a suitable choice for investigations of the so-called tissue culture-induced variation (TCIV), which is demonstrated at the level of biochemical pathways and cycle fluctuations as well as at the level of DNA methylation changes [3] and sequence variation [4] maintained by regenerants. The phenomenon is linked to numerous abiotic stresses applied for the switch from gametophytic to sporophytic path required in anther cultures, cold stress, and darkness [5,6] are the most common. The shift may proceed via direct or indirect somatic embryogenesis and may affect green plant regeneration efficiency (GPRE) [7]. Recent studies demonstrated that TCIV, fluctuations in biochemical pathways and cycles [8], and GPRE could be controlled by Cu(II) ion concentration in the induction medium (IM) [9]. It was shown that under increasing Cu(II) ion concentration, the GPRE is the highest [10], whereas TCIV seems to be the lowest [4].

Furthermore, other cations may be essential. A good example is Ag(I), which influences efficiency of callus formation [11]. The other instances are Zn(II), Fe(II), and Mn(II), participating in anther cultures as ingredients of the in vitro medium and acting as cofactors in biochemical reactions [12,13,14,15]. If they are in shortage or excess, biochemical cycles and pathways might be out of balance, leading to fluctuations of metabolomic compounds [16,17,18] and propelling complex epigenetic machinery [19,20,21]. Among the metabolites that were described as putative participants of the machinery and influenced by metal ions in the in vitro medium, glutathione (GSH) [9], S-adenosyl-L-methionine (SAM) [22], and β-glucans [8] were described.

It was shown that GSH used for pre-treatment of spikes or added to the IM in triticale [9] and rye [23] increased the number of green versus albino plants. GSH is controlled by Cu(II) ion concentration in the IM and seriously impacts GPRE, as shown in triticale anther culture models [9]. It may serve as an antioxidant [24], contribute to the cell homeostasis under toxic metal stress [25], be involved in epigenetic regulation of gene expression [26], and may protect DNA against point mutations originating from methylated cytosines. Multiple GSH functions may advocate its putative epigenetic impact on GPRE [27].

SAM is a byproduct of the Yang cycle which combines ATP from the electron transfer chain (ETC) and L-methionine [28,29]. The cytochrome IV complex, which has Cu(II) ions in its core, catalyzes the creation of ATP [30]. Therefore, SAM production may be stuck if the complex is not operating correctly. In addition, SAM acts as a cellular methylating agent, modifying DNA and histones and other cellular compounds [31]. It may also influence polysaccharide methylation [32]. The data described in humans showed that the excess of SAM may be catabolized to adenine and methylthioadenosine, toxic methylation inhibitors [31]. The significance of SAM for de novo DNA methylation altering the GPRE of triticale anther cultures was highlighted using the structural equation model (SEM) [22]. As methylating agent SAM may participate in gene expression regulation [33] during microspore shift from gametophytic to sporophytic state [34] influencing GPRE [22].

Another metabolic compound that may contribute to GPRE is β-glucan. In *Brassica napus* [35] and rye [36] microspores, β-glucans reside in the cell wall and form the subintinal callose layer [37]. Its present seems to be linked to the embryogenic competence of microspores [38]. It was proposed that β-glucans might serve as the sole source of carbon under tissue culture carbon shortage, cold therapy, and darkness [39] conditions. The idea is backed by the facts that starch is not formed in darkness, and cellulose found in cell walls is highly recalcitrant and thus not efficiently degraded to sugars by enzymes [40], and finally, it is rarely accessible as a source of carbon. Therefore, in the lack of digestible carbs, β-glucans may be the only carbon converted to glucose as a source of energy supply for glycolysis [41,42]. It is possible that the cold treatment of spikes disrupts the cell wall in some way, allowing β-glucans to secrete into the cytoplasm. Glycolysis, utilizing β-glucans, drives the Krebs cycle and ATP synthesis [43]. Recently, it was demonstrated, at least in barley anther cultures, that β-glucans might be involved in GPRE [8].

Moreover, in parallel to β-glucans, the plant’s primary cell wall pectins may be vital for the cell’s functioning, including cell adhesion [44,45] and cell fate specification [46]. Under some conditions (i.e., a limited number of divalent cations, de-esterification), pectins become more soluble, which weakens the connection between the cells and results in cell proliferation [44,47,48,49]. Some studies have demonstrated that pectins participate in the synthesis of ascorbic acid (AA) [50], which acts as an antioxidant under stressful conditions by scavenging reactive oxygen species (ROS). AA, in parallel to GSH in cell suspension, is a signaling molecule involved in the control of plant growth and development, modulating progression through the mitotic cell cycle. ROS accumulation or decreased levels of AA (or GSH) arrests and halts the G1 checkpoint [51,52]. The AA (vitamin C) activates pyruvate dehydrogenase (PDH), encompassing the pyruvate dehydrogenase complex (PDC). The PDC catalyzes the oxidative decarboxylation of pyruvate to release acetyl-CoA (and NADH), targeting the mitochondrial tricarboxylic acid (TCA) cycle in the case of some cancer diseases [53] with similar action on the TCA in rice [54]. A growing body of evidence shows that pectins in the cell wall are localized in spatially restricted patterns. It is becoming recognized that their non-uniform distributions may contribute to the morphogenesis of cells and organs. Distinct contributions of varying pectin fractions or pectin modifications may affect the plant wall stiffness, which may also depend on tissue [44]. An example is the methylesterification of homogalacturonan (HG). In pollen tubes, HG de-methylesterification stiffens the cell wall [55], whereas in meristems and leaves the same process limits wall stiffness [56]. It should be mentioned that pectins participate in plant responses to abiotic stresses [46].

Despite the fact that Cu(II), GSH, and SAM were shown to affect TCIV and GPRE in triticale [9,22,27] and that β-glucans (or other carbohydrates) affect GPRE in barley [8], there is no evidence linking all the factors explaining GPRE in the form of a theoretical model reflecting biochemical and epigenetic levels of the phenomenon in triticale.

We hypothesize that Cu(II) ions in the IM via cytochrome *c* complex IV may affect SAM synthesis via the transsulfuration pathway, they affect GSH, which is involved in the GSH-ascorbate cycle [57,58], and that GSH and SAM influence each other, affecting GPRE. Moreover, SAM synthesis is controlled by glycolysis, whose functioning requires a carbon source. Due to anther tissue culture conditions (darkness, cold treatment, and carbon starvation), β-glucans could be the only source of carbon available for pumping the TCA. Alternatively, carbohydrates, i.e., pectins present in the cell wall or those originating from the Golgi apparatus and transported via cytosol in estrificated form to the wall [59], may also be influential for the Krebs cycle via Acetyl-CoA [54].

The study aims at investigating the relationships between GSH, SAM, and polysaccharides (β-glucans, or pectins) controlled by copper ions in the in vitro medium on anther culture regenerants derived under varying cation ion concentrations in the IM, utilizing FTIR spectroscopy and structural equation modeling to evaluate a theoretical model of GPRE.

## 2. Materials and Methods

### 2.1. Plant Material

The evaluation of plant materials was described elsewhere [4,9]. Briefly, seeds of winter triticale (X *Triticosecale* spp. Wittmack ex A. Camus 1927) cultivar T28/2 derived from cv. Presto × cv. Mungis cross was used for preparing donor plants employing in vitro cultures, and the generative cycle has been described previously [27].

The donor plants served as a source of explants for anther cultures. The regenerants were made by trying different amounts of copper (Cu(II)) and silver (Ag(I)) ions in the IM and different lengths of time for the anther to be incubated on the IM. The induction medium 190-2 [60] with 90 g L^−1^ maltose and 438 mg L^−1^ glutamine supplemented with 2 mg L^−1^ 2,4-dichlorophenoxyacetic acid and 0.5 mg L^−1^ kinetin; the regeneration medium 190-2 [60] supplemented with 0.5 mg L^−1^ naphthalene acetic acid and 1.5 mg L^−1^ kinetin; and the rooting medium N6I [61] supplemented with 2 mg L^−1^ indole-3-acetic acid was implemented. Copper and silver ions were added as salts: CuSO_4_ × 5H_2_O at 0.1, 5, 10 µM, and AgNO_3_ at 0, 10, and 60 µM concentrations. The incubation times were 35, 42, and 49 days, covering the time from plating anthers on IM to calli collection and transferring them onto regeneration media. Eight (A-H) trial conditions were used. For each trial, the number of green regenerants per 100 plated anthers was counted and called “green plant regeneration efficiency” (GPRE).

### 2.2. Infrared Spectroscopy

The Attenuated Total Reflectance–Fourier Transfer Infrared (ATR-FTIR) spectroscopy was applied to inspect lyophilized and homogenized leaf samples as described in our previous papers [9,22]. Briefly, the measurements were conducted using an iZ10 spectrometer equipped with the ATR accessory. The sample was placed on the diamond crystal’s surface and pressed with a clamp to get optimal contact of sample with the crystal. The 64 spectra collected at 4 cm^−1^ resolution were averaged, baseline corrected, and normalized to the unit area within the 1800–900 cm^−1^ wavenumber region using the OMNIC software (v.9.0) and ChemoSpec [62] package in the R programming language [63]. For resolving overlapped peaks, deconvolution was performed using the Gaussian function and nonlinear least-squares fitting [64]. The absorbance integrated within 10 cm^−1^ intervals was used as the input for model analysis.

### 2.3. Statistics

Pearson’s correlations were conducted in SPSS v.28 [65]. SEM, including model characteristics, was performed in SPSS v.28 using AMOS v.27 [66].

## 3. Results

The plant material was described in our earlier studies [4,9]. Briefly, a randomly chosen progeny plant selected from twenty-four double haploid plants uniform in morphological traits (height, leaf size, tillering, and seed set) was used as a donor of explant tissue. Several trials (A-H) differing in Cu(II), Ag(I) ion concentrations in the induction medium (IM), and time of in vitro anther cultures resulted in thirty-seven morphologically uniform regenerants identical with the donor plant. Each trial consisted of 3–10 regenerants. The GPRE was the lowest value in A and the highest in H (Table 1).

**Figure 1:**
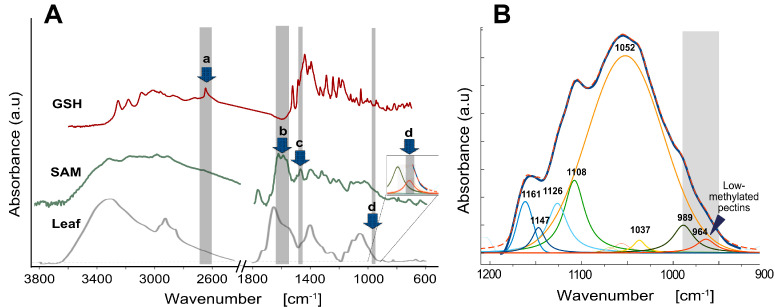
Infrared spectra used for the structural equation model (SEM). (**A**) Combined spectra of the glutathione in reduced form (GSH) and the S-adenosyl-L-methionine (SAM) used as standards and the average spectrum of leaves from tissue culture (leaves). Arrows indicate specific for GSH (a), SAM (b, c) and carbohydrate (d) spectra regions taken as the inputs for the model. The inset shows zoomed the (d) spectral region. (**B**) Results of numerical (Gaussian) deconvolution of overlapping peaks in the IR spectral region of the carbohydrate fingerprint. The experimental data (solid black line) and the data from the sum of fitted curves (dotted red line) were very close to each other. The centers of spectral bands re-solved through the deconvolution are marked with wavenumbers. The region relevant to the proposed model is shaded grey and corresponds to the (d) region in the (**A**) panel.

Some of the spectral regions were given to the different metabolic compounds based on what we had learned from studying the reference chemical compounds. For example, the signal from the reduced form of glutathione (GSH) is specifically located at 2550–2540 cm^−1^ and attributed to the S-H stretching vibrations [9] (Figure 1A). In turn, the S-adenosyl-L-methionine was tentatively linked to the combined two ranges of 1630–1590 and 1490–1470 cm^−1^ (Figure 1A).

Searching for carbohydrate compounds that could drive the Krebs cycle, we focused on carbohydrate spectral region between 1200–900 cm^−1^. First, we considered IR spectra between 1180–1160 cm^−1^ characteristic of β-glucan. However, there was no variation in the region’s absorbance among samples (as well as trials). Further, we used spectrum deconvolution of the carbohydrate band within 1200–900 cm^−1^ wavenumbers using gaussian band shapes and an iterative curve fitting procedure (Figure 1B) to detect constituent components comprising the complex band. The region within 990–950 cm^−1^ was found to contribute most significantly to the model. However, it was not assigned clearly to any metabolic compounds.

The IR spectra ranges reflecting GSH [9], SAM [22], and the unassigned 990–950 cm^−1^ region, as well as the respective tissue culture conditions (Cu(II), Ag(I), and time) used for plant regeneration, were implemented as variables explaining GPRE in SEM analysis. The model was built on 37 samples. Skewness and kurtosis values observed a minor deviation from the normal distribution (Table 2). All quantitative variables met the Lindeberg–Lévy theorem’s [67] assumptions. Thus, the variables asymptotically converged with the theoretical distribution. The maximum likelihood option was used for the postulated model’s construction.

The highest Pearson positive correlation values were between Cu(II) and GPRE, followed by the correlation between GSH and SAM, GSH with GPRE, Cu(II) and GSH, and ending with Cu(II) and GSH. All the correlations except [F990_950] and time were positive. Ag(I) was not correlated with any of the variables. The other correlations were insignificant (Table 3).

**Figure 2:**
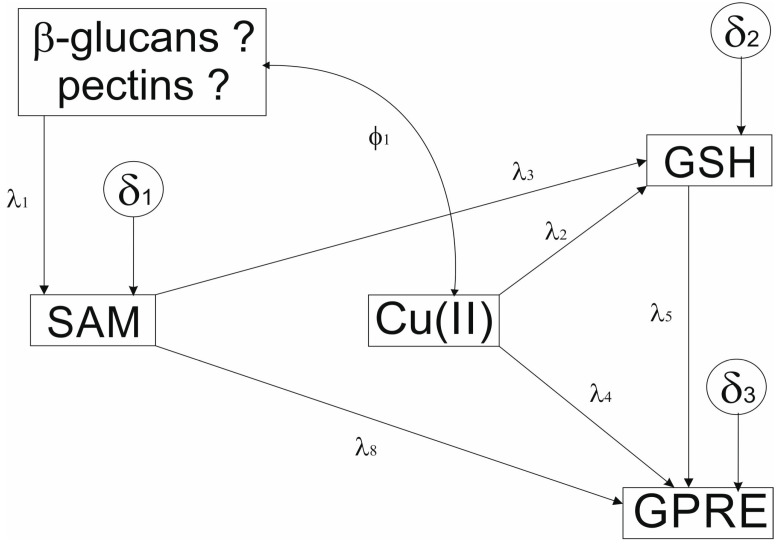
The hypothesized SEM model illustrates relationships between Cu(II), SAM, and GSH, and [990_950] explaining GPRE. GPRE-green plant regeneration efficiency (number of regenerants per 100 plated anthers); GSH, SAM, and β-glucans/pectins denote contribution from IR spectrum absorbance for wavenumber ranges of 2550_2540 cm^−1^, 1630…1470 cm^−1^ and 990_950 cm^−1^. *λ*1-*λ*6 path coefficients, *δ*1-*δ*3 residuals (experimental errors). According to the model, carbohydrates (b-glucans/pectins) pump the Krebs cycle affecting the Yang cycle and influencing SAM. SAM, via GSH, indirectly influences GPRE. It also has a direct effect on GPRE. Copper ions act as cofactors of enzymatic reactions modifying biochemical reactions and influencing GPRE.

The postulated model has two exogenous variables (Cu(II) and [990_950]) and three endogenous (GSH, SAM, and GPRE). All relationships were non-recursive. The covariance between Cu(II) and [990_950] was insignificant, as indicated by the lack of correlation between the variables (Table 3). All but the Cu(II) variables were observed. The model included three residuals (Figure 2).

Analysis of the so-called ad hoc fit indices showed that the *χ^2^* statistics (Table 4) of the model fitting were insignificant. So, it was used as an information criterion [68] because the small sample size used to build models could lead to an incorrect model being accepted [69]. Thus, the other goodness-of-fit models’ descriptive characteristics were evaluated, including the *χ^2^*/df one. Its value was less than 3, which shows that the proposed model fits the data. The goodness-of-fit measures (RMR, SRMR, GFI, AGFI, and PGFI) were within the suggested ranges [70] (Table 4). The same is valid for the comparative indices of fit (NFI, RFI, IFI, TLI, and CFI) which exceeded 0.95 in all but one (RFI) case. Furthermore, the RMSEA index was below 0.05. The RMSEA is below 0.05, and the probability value associated with this test of close fit is above 0.5 (see PCLOSE). The low values of the parsimony indices show that the model is complex. However, as most of the statistics fell within the expected limits, the postulated model fit well with the experimental data.

The postulated model’s paths’ (*β*) coefficients were significant (Table 5). The highest positive effects were observed for the Cu(II) and GPRE path, followed by SAM, GSH, and GSH on GPRE. The only adverse effect was that of SAM on GPRE.

Cu(II) positively influenced GPRE primarily via the direct effect (*β* = 0.8105), whereas the indirect effect was relatively small (*β* = 0.1594), demonstrating the significance of the direct effect (*β* = 0.6511) (Table 6). GPRE was also directly affected by GSH exclusively via direct effect (*β* = 0.4638). On the other hand, SAM affected GSH solely via a positive direct effect (*β* = 0.6339). Cu(II) exhibited an intermediate direct effect on GSH (*β* = 0.3437), whereas the [990_950] FTIR variable had the lowest indirect effect on glutathione (*β* = 0.2462). The [990_950] variable exhibited a wholly direct effect on SAM.

## 4. Discussion

Plant material homogeneity is demonstrated by the absence of morphological differences between donor plants and regenerants (at least at the morphological level). Triticale typically has no visual abnormalities, and morphological deviations hardly ever occur in this plant [3]. However, it does not rule out the possibility of common mobile element migration, sequence variation, and changes in DNA methylation patterns. Evidence suggests that variation induced via tissue culture affects both gene expression [71] and biochemical [72] levels. While research on TCIV was reported in a number of studies on barley [73] and triticale [4,74], the impact of short RNAs and alterations in gene expression has been mostly studied in relation to plant regeneration in anther culture [75]. The same is somewhat true for research that reflects biochemical levels. The epigenetic background of TCIV and GPRE may be affected by biochemical cycles and pathways, as has been demonstrated for various cereals [8,9,22]. The problem is crucial because understanding the biology of TCIV and GPRE may have both scientific and practical implications.

Based on previous studies, we have demonstrated that GPRE in triticale depends on the cellular SAM [22], GSH [9] and Cu(II) ions of in vitro tissue culture medium [4,27]. The results have an evident biochemical background reflecting the role of Cu(II) in the ETC [30], in the Yang cycle [76], the transsulfuration pathway [77], and an apparent linkage to copper-mediated DNA methylation changes and mutations [78]. Comparable analysis in barley [7] showed that β-glucans present in between the cell wall and cell membrane might serve as a source of carbon pumping the Krebs cycle via glycolysis [39,79]. If β-glucans are accessible for glycolysis, then the Krebs cycle may function properly, producing ATP required in the Yang cycle for SAM production. However, ATP synthesis is controlled by Cu(II) ions encompassing active center of cytochrome *c* complex IV. If Cu(II) ions in the cell are not balanced SAM synthesis may be distorted. In consequence, the transsulfuration pathway leading to GSH is affected. Furthermore, GSH functioning requires Cu ions. Both GSH and SAM are involved in the complex regulation of epigenetic mechanisms that may affect GPRE. The later notion was confirmed in studies on triticale anther culture regenerants where relationships between the two metabolites and TCIV and GPRE were evaluated [9,22]. Alternatively, the Krebs cycle could be affected by pectins [54], omitting glycolysis. However, little is known on pectins in triticale, whereas β-glucans are most abundant in walls of the cereals, including rye and oats [80,81], and to lesser extent in wheat [82] grains. They may be also present in the secondary wall of certain tissues in the Poaceae [41]. It cannot be excluded that cellulose may also participate in GPRE. However, due to its insolubility, it would be hardly bizarre if such a situation took place. The data mentioned above suggest that the model explaining GPRE in triticale may encompass, i.e., SAM, GSH, either β-glucans or pectins and Cu(II). Thus, evidence for either β-glucans or pectins (or other metabolites) was needed to build the putative relationships between numerous factors affecting GPRE.

The assignation of the band to 1-3, 1-4 mixed glucans was inferred on the basis of numerical deconvolution of the carbohydrate fingerprint, where a strong signal was observed from a component at around 1070 cm^−1^ that may be reasonably linked with a strong peak in the β-glucan spectrum [83], which reflects the C–O and C–C stretching vibrations. However, in the current study, analyzing FTIR spectra, we have failed to detect a characteristic band within 990-950 cm^−1^ region of the carbohydrate fingerprint attributed to β-glucans [7,8]. The deconvolution performed on the spectra of triticale, in the present study, did not generate the expected signal at around 1070 cm^−1^. Instead, we observed a strong component at 1052 cm^−1^, tentatively attributed to cellulose [84]. The cellulose may also contribute to the 990–950 cm^−1^ region through absorbance of the massive peak shoulder. However, it cannot be considered a credible carbon source for biochemical reactions as cellulose is insoluble [85] and cannot be easily utilized by the cell. We have also failed to find evidence suggesting that cellulose may contribute either directly or indirectly to the Krebs cycle. Furthermore, the 990–950 cm^−1^ absorbance may also be related to polygalacturonic acid (PGA), particularly in highly demethylated form [86]. Thus, the absorbance in the area could be related to differences in the level of methylesterification of pectins in the cell wall, which Cu(II) ion treatments could change during the in vitro culture. This may affect the regeneration processes as pectins demethylation may reorganize cell wall structure indirectly [87] or directly [88]. The control of growth symmetry breaking in the Arabidopsis hypocotyl [89,90] affects the cell wall expansion, and thus growth driven by turgor, and is possibly involved in morphogenesis via local wall expansion due to swelling of the HG nanofilaments [91]. Pectins are common to triticale in contrast to barley, where β-glucans predominate [92,93]. Furthermore, via PDH, pectins may indirectly influence the Krebs cycle [54] affecting its functioning under varying conditions. The presented reasoning convinced us that the most probable metabolites participating in the relationships between SAM, GSH, Cu(II), and GPRE are pectins rather than β-glucans. However, it is not apparent whether pectins originate from the primary wall of the cells or are the fraction from the Golgi apparatus.

The FTIR spectra for pectins, SAM, and GSH, as well as the Cu(II) ion concentration in the IM (Ag(I) ion concentration and time of another culture were also tested), were put into a structural equation model using a specially designed biological system that included regenerants from a single donor plant grown under various in vitro culture conditions (Table 1). With a small sample size, the goodness-of-fit indices could be a long way outside the anticipated boundaries. Statistics, however, showed that the theorized model was a good fit for the experimental data. However, the study’s most glaring drawback is its small sample size. In anther culture, it takes much work to bring back many plants and get a sample big enough for analysis.

In addition, the process is limited by the presence of albino plants, which could make GPRE less effective. On the other hand, the low number of regenerants in each trial is not surprising, given that a single donor plant, a generative progeny of DH, was used as a source of explant tissue for as many as eight trials. Our findings indicate that, in trials, a particular number of regenerants could be assessed. Therefore, we think the differences are caused by the tissue culture and not by chance, even though the problem needs to be looked into more. Analysis in barley [7] showed a similar variance in the number of regenerants, which may further corroborate the idea that culture conditions impact GPRE.

Analysis of the variables used in the model showed no apparent problems with their normal distributions, which is why they were used. Moreover, the correlations showed relationships between them, which is a requirement for building an SEM. A detailed analysis of paths confirmed our hypothesis concerning relationships between variables and GPRE. The model was based on what we know about the biochemical background of how well anther culture plants can grow back. The most exciting finding is that SAM affects GSH. This path is the second most important positive effect of the model. Studies on rye [94] and triticale [23] treated with GSH showed that its presence positively affected plant regeneration. The effect was observed independently of whether GSH was used as a pre-treatment [23,94] or was added to the IM [95]. Thus, our results are fully congruent with those data.

Interestingly, pectins implemented in the model demonstrated that they positively affected SAM synthesis. The impact of pectins relies on their indirect action on the Krebs cycle. The presented data differ from those for barley anther cultures [8] where β-glucans were suggested to participate in the model. We have also proposed that Cu(II) ions acting as cofactors of enzymatic reactions are vital players in anther cultures. The notion is evidenced by the most robust path evaluated for Cu(II) on GSH.

Furthermore, Cu(II) also positively affected GPRE. Interestingly, the model did not find Cu(II) on the SAM path, which is what would have been expected if Cu(II) worked as a cofactor for cytochrome *c* complex IV. Nevertheless, the parsimony indices showed that the model was too complicated, and the sample size used to build the model was too small to find such an effect.

An interesting aspect of the presented model is the fact that covariance between Cu(II) and pectins is non-significant. Furthermore, the two variables are not correlated. It should be stressed, however, that Cu(II) and pectins remained exogenous variables. While it is not unexpected for Cu(II) as its concentration was manipulated experimentally, it is not easy to explain why pectins had to be treated as exogenous variable too. The alternative models with pectins being treated as the endogenous variable failed to fit experimental data (not shown). We tend to speculate that there must be another variable not implemented in the model that controls pectins. Further studies are required to verify the presented model.

## 5. Conclusions

In conclusion, the data show the connections between SAM, GSH, pectins, and Cu(II) in the IM and how they affect GPRE. The SEM model reflects crucial aspects of the cell functioning under in vitro conditions and varying Cu(II) concentrations. The Krebs, the Yang cycles, the transsulfuration pathway controlled by Cu(II) ions acting as cofactors of enzymatic reactions, and the pectins of the primary cell wall are the players of the presented model.

## Figures and Tables

**Table 1:** The arrangement of the induction medium composition and the time of anther culture contraposed with GSH, SAM and β-glucans FTIR spectra explaining GPRE via structural equation model.

Trial	In Vitro Anther Culture Conditions			GSH ^1^ (2550_2540 cm^−1^)	SAM (1630_1590 + 1490_1470 = 1630…1470 cm^−1^)	β-Glucans?/ Pectins? (990_950 cm^−1^)	GPRE
	Cu (μM)	Ag (μM)	Time (Days)				
A	0.1	10	42	0.004591	3.40998	0.42449	0.87
	0.1	10	42	0.004940	3.68581	0.46012	0.87
	0.1	10	42	0.005324	4.25217	0.42903	0.87
B	0.1	60	49	0.004879	3.2412	0.53629	1.52
	0.1	60	49	0.005120	3.99229	0.70485	1.52
	0.1	60	49	0.005144	3.96066	0.63372	1.52
	0.1	60	49	0.004353	3.26579	0.54054	1.52
	0.1	60	49	0.004985	3.84068	0.52415	1.52
C	5	60	42	0.004768	4.32409	0.48983	0.71
	5	60	42	0.005038	4.26221	0.51636	0.71
	5	60	42	0.004120	3.35044	0.39522	0.71
D	5	0	49	0.005176	4.34178	0.59499	2.38
	5	0	49	0.005264	4.22150	0.54191	2.38
	5	0	49	0.005502	3.97269	0.35829	2.38
	5	0	49	0.006040	4.60325	0.61184	2.38
	5	0	49	0.005451	4.01752	0.47999	2.38
	5	0	49	0.005293	3.98898	0.41253	2.38
	5	0	49	0.005133	4.26056	0.48079	2.38
	5	0	49	0.005520	4.61482	0.54557	2.38
	5	0	49	0.004793	4.18367	0.55962	2.38
	5	0	49	0.005358	4.54641	0.71383	2.38
E	5	10	35	0.004881	4.24147	0.63052	1.17
	5	10	35	0.004362	3.52101	0.59939	1.17
	5	10	35	0.004652	3.75418	0.70079	1.17
	5	10	35	0.005249	4.08202	0.62406	1.17
	5	10	35	0.005302	4.18035	0.94729	1.17
F	10	10	49	0.005046	3.31130	0.42781	3.79
	10	10	49	0.005121	3.33494	0.43148	3.79
	10	10	49	0.005566	3.64536	0.47079	3.79
G	10	60	35	0.005394	4.52669	0.69809	4.24
	10	60	35	0.005685	4.41428	0.70797	4.24
	10	60	35	0.005823	4.65225	0.68606	4.24
	10	60	35	0.005085	3.92419	0.66115	4.24
H	10	0	42	0.005692	4.26782	0.45602	6.06
	10	0	42	0.005502	3.86516	0.44444	6.06
	10	0	42	0.004594	2.65280	0.51390	6.06
	10	0	42	0.005502	3.87212	0.44032	6.06
Means				0.005142	3.961688	0.551190	2.56
SD				0.000425	0.463502	0.122311	1.66

^1^ GSH-glutathione; SAM-S-adenosyl-L-methionine; A–H—trials with different in vitro conditions; GPRE—states for green regenerants obtained per 100 plated anthers; SD—standard deviation

**Table 2:** Descriptive statistics of the variables present in the postulated models.

Variables	Mean	SD ^1^	Variance	Skewness	Kurtosis
[Cu(II)]	5.4270	3.5759	12.787	−0.102	−1.008
[Ag(I)]	22.4324	26.7089	713.363	0.704	−1.480
[Time]	43.7027	5.8112	33.770	−0.495	−1.371
[1630...1470]	3.9617	0.4635	0.215	−0.709	0.278
[2550_2540]	0.0051	0.0004	0.000	−0.351	0.054
[990_950]	0.5512	0.1223	0.015	0.951	1.406
[GPRE]	2.5563	1.6589	2.752	0.932	−0.122

^1^ SD-standard deviation.

**Table 3:** Pearson’s linear correlation coefficients for analyzed variables.

Variables	[Cu(II)]	[Ag(I)]	[Time]	[1630...1470]	[2550_2540]	[990_950]	[GPRE]
[Cu(II)]	1						
[Ag(I)]	−0.181	1					
[Time]	−0.312	−0.203	1				
[1630...1470]	0.075	−0.020	−0.116	1			
[2550_2540]	0.385 *	−0.229	0.081	0.650 **	1		
[990_950]	0.009	0.252	−0.451 **	0.388 *	0.137	1	
[GPRE]	0.807 **	−0.201	−0.061	−0.052	0.461 **	−0.117	1

*. Correlation is significant at the 0.05 level (2-tailed). **. Correlation is significant at the 0.01 level (2-tailed).

**Table 4:** Summary of the analyzed structural equation model.

	Statistics	Goodness-of-Fit Statistics
Ad hoc indices of fit	Chi-square (*χ^2^*)	1.4658
	Degree of freedom (df)	3
	*p*-value (*p*)	0.6902
	CMIN(*χ^2^*)/df	0.4886
	Root Mean Square Residual (RMR)	0.0354
	Standardized RMR (SRMS)	0.0448
	Goodness-of-fit index (GFI)	0.9842
	Adjusted Goodness-of-fit Index (AGFI)	0.9211
	Parsimony GFI (PGFI)	0.1968
Comparative or incremental indices of fit	Normed Fit Index (NFI)	0.9833
	Relative Fit Index (RFI)	0.9443
	Incremental Fit Index (IFI)	1.0181
	Tucker-Lewis Index (TLI)	1.0658
	Comparative Fit Index (CFI)	1
Model parsimony	PRATIO	0.3
	PNFI	0.295
	PCFI	0.3
Error approximation index	Root Mean Square Error of Approximation (RMSEA)	0
	PCLOSE	0.7194
	Expected Cross-Validation Index (ECVI)	0.7074
	Hoelter’s Critical N (0.5)	192

**Table 5:** Path coefficients, variances and covariances for the analyzed model.

Parameter	Effect			Estimate (*b*)	Standard Error	Test Statistic	Standardized Estimate (*β*)
*Path coefficients*							
*λ*_1_	[990_950]	→	[1630…1470]	1.4717	0.582	2.5287 *	0.3884
*λ*_2_	[Cu(II)]	→	[2550_2540]	0	0	2.9726 **	0.3437
*λ*_3_	[1630...1470]	→	[2550_2540]	0.0006	0.0001	5.4822 ***	0.6339
*λ*_4_	[Cu(II)]	→	[GPRE]	0.304	0.0416	7.3094 ***	0.6511
*λ*_5_	[2550_2540]	→	[GPRE]	1850.4849	459.0907	4.0308 ***	0.4638
*λ*_6_	[1630...1470]	→	[GPRE]	−1.4651	0.3895	−3.7613 ***	−0.4067
*Covariances*							
	Cu(II)	←→	[F990_950]	0.0038	0.0709	0.0536	
*Variances*							
*δ*_1_				0.1775	0.0418	4.2426 ***	
*δ*_2_				0	0	4.2426 ***	
*δ*_3_				0.6188	0.1458	4.2426 ***	
[Cu(II)]				12.4414	2.9325	4.2426 ***	
[990_950]				0.0146	0.0034	4.2426 ***	

*—significant at *p* ≤ 0.05; **—significant at *p* ≤ 0.01; ***—significant at *p* ≤ 0.001

**Table 6:** Direct, indirect and total effects for the analyzed model.

Effect			Estimates (*b*)			Standardized Estimates (*β*)		
			Direct Effect	Indirect Effect	Total Effect	Direct Effect	Indirect Effect	Total Effect
[GPRE]								
[990_950]	→	[GPRE]	0	−0.598	−0.598	0	−0.044	−0.044
[Cu(II)]	→	[GPRE]	0.304	0.0744	0.3785	0.6511	0.1594	0.8105
[1630…1470]	→	[GPRE]	−1.465	1.059	−0.406	−0.407	0.294	−0.113
[2550_2540]	→	[GPRE]	1850.5	0	1850.5	0.4638	0	0.4638
[2550_2540] (GSH)								
[990_950]	→	[2550_2540]	0	0.0008	0.0008	0	0.2462	0.2462
[Cu(II)]	→	[2550_2540]	0	0	0	0.3437	0	0.3437
[1630…1470]	→	[2550_2540]	0.0006	0	0.0006	0.6339	0	0.6339
[1630…1470] (SAM)								
[990_950]	→	[1630…1470]	1.4717	0	1.4717	0.3884	0	0.3884

## Data Availability

Not applicable.

## Abbreviations

AA	Ascorbic Acid
ETC	Electron Transport Chain
FTIR	Fourier Transform Infrared
GPRE	Green Plant Regeneration Efficiency
GSH	Glutathione
HG	Homogalacturonan
IM	Induction Medium
PDC	Pyruvate Dehydrogenase Complex
PDH	Pyruvate Dehydrogenase
ROS	Reactive Oxygen Species
SAM	S-Adenosyl-L-Methionine
SEM	Structural Equation Model
TCA	Tricarboxylic Acid
TCIV	Tissue Culture-Induced Variation
